# Impact of Vitamin D Supplementation on Arterial Vasomotion, Stiffness and Endothelial Biomarkers in Chronic Kidney Disease Patients

**DOI:** 10.1371/journal.pone.0091363

**Published:** 2014-03-19

**Authors:** Nihil Chitalia, Tuan Ismail, Laura Tooth, Frances Boa, Geeta Hampson, David Goldsmith, Juan Carlos Kaski, Debasish Banerjee

**Affiliations:** 1 Renal and Transplantation Unit, St George's Healthcare NHS Trust, London, United Kingdom; 2 Cardiovascular Research Centre, St George's, University of London, London, United Kingdom; 3 Chemical Pathology, St George's Healthcare NHS Trust, London, United Kingdom; 4 Renal and Clinical Chemistry Departments, Guy's and St Thomas, NHS Foundation Trust, London, United Kingdom; Indiana University Richard M. Fairbanks School of Public Health, United States of America

## Abstract

**Background:**

Cardiovascular events are frequent and vascular endothelial function is abnormal in patients with chronic kidney disease (CKD). We demonstrated endothelial dysfunction with vitamin D deficiency in CKD patients; however the impact of cholecalciferol supplementation on vascular stiffness and vasomotor function, endothelial and bone biomarkers in CKD patients with low 25-hydroxy vitamin D [25(OH)D] is unknown, which this study investigated.

**Methods:**

We assessed non-diabetic patients with CKD stage 3/4, age 17–80 years and serum 25(OH)D <75 nmol/L. Brachial artery Flow Mediated Dilation (FMD), Pulse Wave Velocity (PWV), Augmentation Index (AI) and circulating blood biomarkers were evaluated at baseline and at 16 weeks. Oral 300,000 units cholecalciferol was administered at baseline and 8-weeks.

**Results:**

Clinical characteristics of 26 patients were: age 50±14 (mean±1SD) years, eGFR 41±11 ml/min/1.73 m^2^, males 73%, dyslipidaemia 36%, smokers 23% and hypertensives 87%.

At 16-week serum 25(OH)D and calcium increased (43±16 to 84±29 nmol/L, p<0.001 and 2.37±0.09 to 2.42±0.09 mmol/L; p = 0.004, respectively) and parathyroid hormone decreased (10.8±8.6 to 7.4±4.4; p = 0.001). FMD improved from 3.1±3.3% to 6.1±3.7%, p = 0.001.

Endothelial biomarker concentrations decreased: E-Selectin from 5666±2123 to 5256±2058 pg/mL; p = 0.032, ICAM-1, 3.45±0.01 to 3.10±1.04 ng/mL; p = 0.038 and VCAM-1, 54±33 to 42±33 ng/mL; p = 0.006. eGFR, BP, PWV, AI, hsCRP, von Willebrand factor and Fibroblast Growth Factor-23, remained unchanged.

**Conclusion:**

This study demonstrates for the first time improvement of endothelial vasomotor and secretory functions with vitamin D in CKD patients without significant adverse effects on arterial stiffness, serum calcium or FGF-23.

**Trial Registration:**

ClinicalTrials.gov NCT02005718

## Introduction

Patients with chronic kidney disease (CKD) have up to 3.5 fold increased risk of cardiovascular (CV) events and cardiovascular disease (CVD) is the most common cause of mortality and morbidity [1]. Evidence from general population surveys has shown that CKD is an independent risk factor for CVD [2].

Vascular atherosclerosis is a predominant cause of CV events in CKD, with endothelial cell activation as its primary inciting event [3], [4]. Endothelial dysfunction is the final common pathway of vascular injury mediated by both traditional and non-traditional risk factors of CVD [5]. Circulating biomarkers of endothelial cell activation such as Intracellular Adhesion Molecule (ICAM), Vascular Adhesion Molecule (VCAM), Endothelial leucocyte adhesion molecule (E-Selectin) and platelet adhesion molecule, such as von willebrand factor (vWF), have been validated for assessment of endothelial function [6]. Flow mediated dilatation (FMD) of the brachial artery in response to distal ischaemia, has been validated for assessment of endothelial function in-vivo [6]. Brachial artery FMD is a surrogate for CV disease, as it correlates with coronary atherosclerosis [7]. FMD has been shown to predict future CV events and mortality in the general population and in patients with mild to moderate CKD [8], [9]. Additionally, it has been used extensively to study the effects of pharmacological therapies on vascular function in-vivo [10]–[12]. More importantly, an improvement in endothelial function, as measured by brachial artery FMD, may relate to better prognosis in patients with atherosclerotic disease [13].

Low vitamin D is associated with endothelial dysfunction and high CV mortality in patients with CKD [14], [15]. Native vitamin D treatment has been shown to improve endothelium mediated vascular responses in experimental studies. Studies on spontaneously hypertensive rats show an improvement in vascular response to acetylcholine following a 6 week treatment with vitamin D [16], [17]. In type II diabetic patients and 25-hydroxy vitamin D [25(OH)D] deficiency, vitamin D2 treatment over 8 weeks significantly improved brachial artery FMD [18]. We have previously reported an association between reduced vitamin D levels and impaired brachial artery FMD in CKD patients [14]. However, it is unknown if vitamin D supplementation is associated with an improvement in endothelial function in patients with CKD.

Serum Fibroblast Growth Factor-23 (FGF-23) a phosphaturic hormone secreted by osteoblasts, inhibits 1 alpha hydroxylase enzyme, preventing conversion of 25 (OH)D to 1,25 dihydroxy vitamin D [1,25 (OH)_2_D] is elevated in CKD, and associated with mortality, coronary artery atherosclerosis and left ventricular hypertrophy [19]–[21]. However, it is not known if nutritional vitamin D treatment in vitamin D insufficient/deficient CKD patients, changes serum FGF-23 levels.

As the impact of Vitamin D supplementation on arterial vasomotor and stiffness function, endothelial and bone biomarkers has not been investigated systematically, this study tested the effect of treatment with oral cholecalciferol on endothelial function, arterial stiffness, biomarkers of endothelial cell activation and serum FGF-23, in relatively healthy non-diabetic CKD stage 3 and 4 patients with low levels of vitamin D.

## Methods

The protocol for this trial and supporting CONSORT checklist are available as supporting information; see Checklist S1 and Protocol S1. The brief methods are as follows.

### 1. Patients

**Figure 1:**
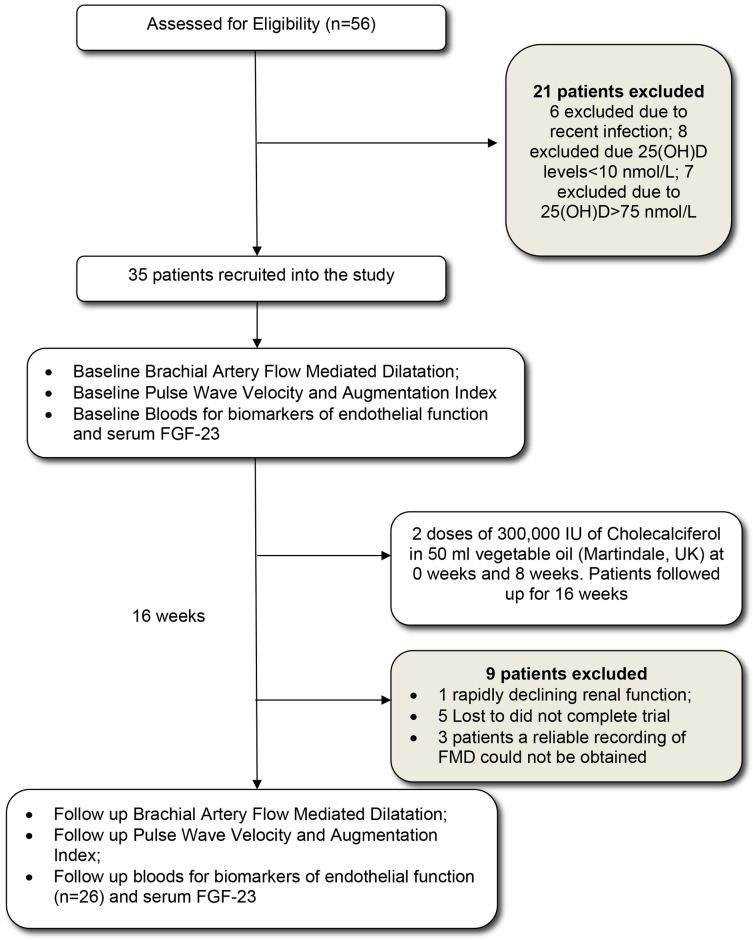
Number of patients recruited into the study and completed the follow-up. Legend: Flow Diagram of the patient pathway recruited into the effects of oral vitamin D on endothelial function intervention study. Thirty five patients were recruited into the study after excluding twenty one patients. Twenty six patients completed the study.

Stable (past 6 months) stage 3–4 CKD patients, with low vitamin D (25(OH)D<75 nmol/L), were recruited. Baseline serum 25 (OH)D were measured during routine clinic visits for patients. Patients were excluded from the study if they were known to have diabetes mellitus, malignancy, heart failure (Ejection fraction on ECHO<40% or N-terminal pro Brain natriuretic peptide (NT-proBNP)>500 pg/ml), active inflammation, active autoimmune conditions, recent acute coronary syndrome (within last 3 months), rapidly deteriorating renal function, corrected serum calcium>2.55 mmol/L and if already on Vitamin D supplementation. Patients were considered to have diabetes mellitus if they were on insulin, oral hypoglycaemic agents, diagnosed as diet controlled diabetes, had a fasting blood sugar ≥7 mmol/L or random glucose ≥11.1 mmol/L. The study was approved by NRES Surrey Research Ethics committee (REC reference 10/H1109/14) and all patients provided written informed consent. Patient recruitment and follow up is represented in figure 1. Patients were recruited between June 2010 and May 2012 follow up completed in September 2012. The trial was registered after study completion to ensure proper conduct and we confirm that all on-going and related trials with Vitamin D are registered.

### 2. Brachial artery FMD

Brachial artery FMD was estimated at baseline and on follow up at 16 weeks. All study subjects were assessed in our vascular laboratory, in a quiet purpose-built room maintained at constant temperature of 22–24°C, after 10 min of rest in the supine position, and after 12 hour overnight fasting. Patients were asked to withhold their regular medications for the day, until after the scan. Caffeine and tobacco were disallowed in the 6 hours prior to the scan. A standard HDI (High Definition Imaging) 3000 ultrasound system (ATL, Bothell, WA, USA) equipped with a 12–5 MHz linear array transducer was used for endothelial function measurements. The same trained physician performed all scans. The overall mean (SD) intra patient variability of this technique within our department is 0.9 (0.48) % (range 0.21–2%) [22].

The right arm brachial artery was scanned in a longitudinal section 2–10 cm above the elbow and its diameter measured continuously with on-line, wall-tracking software for 1 min at baseline, during 4.5 min of induced distal ischaemia (induced by inflation to 300 mmHg of a pneumatic cuff placed at a site distal to the segment of the artery being analysed), and for another 5 min during reactive hyperaemia after cuff release. After return to baseline, vessel diameter was again measured continuously up to 3 min for baseline measurements and for 5 min after administration of 50 µg sublingual glyceryl trinitrate, a direct nitric oxide donor. FMD was defined as the maximum percentage increase in vessel diameter during reactive hyperaemia; endothelium independent FMD was defined as the maximum percentage increase in vessel diameter after sublingual glyceryl trinitrate.

### 3. Pulse wave velocity and Augmentation index

The pulse wave velocity (PWV) was measured at baseline and at week 16. This was done at the same time as the brachial artery FMD. Arterial stiffness was assessed non-invasively using a pressure tonometer coupled to a SphygmoCor PWV system. A simultaneously recorded ECG signal (continuous recording of 3 chest leads) provided an R-wave timing reference. Pulsation from two contra lateral recording sites, the common carotid artery and the femoral artery were identified and marked. The distance between the suprasternal notch and common carotid pulsation was subtracted from the distance between the suprasternal notch and the femoral artery pulse to obtain the PWV distance. A pressure tonometer was then placed at the two recording sites to obtain 10 homogenous continuous waveforms. The software processed each set of pulse waveforms and ECG waveform data to calculate the mean time difference between the arrivals of the pulse at the two peripheral recording sites on a beat to beat basis. The PWV was then calculated using the mean time difference and the arterial path length between the two recording sites. At the same time blood pressure was recorded over the right arm and a mean of three readings was considered. Height (without footwear) and weight (in street clothes) was measured and recorded in the SphygmoCor PWV system. Using the same equipment, the radial pulse waveform was analysed and the corresponding central aortic waveform generated using a transfer function. The augmentation index (AI) was calculated as the ratio of the difference between early and peak systolic pressure versus the early systolic pressure from the derived aortic wave form.

### 4. Measurement of circulating biomarkers

Whole blood samples were obtained in SST tubes during baseline and follow-up scans. The samples were centrifuged immediately at 4000 rpm for 15 minutes and the serum separated. Serum was analysed for baseline 25(OH)D levels using a chemiluminescent immunoassay on an automated IDS-iSYS analyser. The analytical range was 12.5–350 nmol/L and inter-batch co-efficient of variation (CV) <12%. High sensitivity C-reactive protein (hsCRP) was measured using the hsCRP latex-enhanced immunoturbidimetric assay (Siemens Healthcare Diagnostics) on an ADVIA 2400 analyser. The analytical range was 0.16–10 mg/L and the inter batch CV<10%. The remaining serum was stored at −70°C.

Baseline and follow-up serum samples for each patient were measured simultaneously in duplicates for endothelial function biomarkers, with mean value of the two concentrations taken as the representative concentration of the biomarker in the sample.

Serum E-Selectin was measured using ELISA (Abcam). The samples and the standards were diluted 1∶100. The assay range for this ELISA is 1600–50,000 pg/ml and the intra-assay and inter-assay CV% is 5.4 and 6.0%, respectively.

Serum ICAM was measured using ELISA (Thermo Scientific). The samples were diluted in the ratio of 1∶100. The assay range for this ELISA is 0.3–10 ng/ml and the intra-assay CV% is <10%.

Serum vWF was measured using ELISA (Abcam). The samples were diluted in the ratio of 1∶100. The assay range for this ELISA is 3–30 mU/ml and the intra-assay and inter-assay CV% is 5.1 and 7.2%, respectively (note 1 IU/ml = 9.8 µg/ml).

Soluble VCAM-1 (sVCAM-1) was measured using ELISA (Invitrogen). The samples were diluted in the ratio of 1∶50. The assay range for this ELISA is 0–75 ng/ml and the intra-assay and inter-assay CV% is <4.6 and <8.2%.

Serum FGF-23 was measured by second-generation ELISA (Immutopics, Inc, San Clemente, California) detecting both intact FGF-23 and C-terminal fragments. The interassay CV for this assay was 8.6% and 7.8% at plasma concentrations of 34.6 and 280.8 RU/mL, respectively.

### 5. Vitamin D therapy

All patients recruited received 2 doses of oral 300,000 units of Cholecalciferol in vegetable oil (Martindale Pharma) at the beginning of the study and at week 8. This dose of cholecalciferol has been found to be safe and effective in lowering serum parathyroid hormone (PTH) levels in CKD 3/4 patients, without hypercalcemia [23]. A study from our hospital in patients with 25 (OH)D<40 nmol/L, using 300,000 IU of the same preparation showed a significant decline of 25 (OH)D levels at 12 weeks post administration [24], hence we chose to readminister 300,000 units at 8 weeks to maintain a biological effect of vitamin D3.

### 6. Statistical analysis

Data were analysed using Statistical Package for Social Sciences (SPSSv.16, Chicago, IL). Continuous and normally distributed variables are expressed as mean±1SD. Continuous and non-normally distributed variables are expressed as median±Interquartile range (IQR). Categorical variables are expressed as percentages (%). Comparison of means for continuous, parametric variables was performed using dependent sample T-test and for non-parametric variables using Wilcoxon signed rank test. Comparison of categorical variables was performed using Chi-squared testing. A twosided p-value of <0.05 was considered to indicate statistical significance.

### 7. Sample Size Calculation

The sample size was calculated to show a 10% improvement in mean FMD with vitamin D treatment. The mean FMD of patients recruited into our previous study was 3.8%. The standard deviation of the intra-patient variability in FMD measurements, is 0.48% (range: 0.21–2%) [22]. Taking α = 0.05 and a β = 20, twenty-seven patients were required to show a 10% change in FMD measurements. Assuming a dropout rate of 30% due to failure of study follow-up, progression to ESRD, transfer to other renal units or death, 35 patients were recruited into the study.

## Results

Thirty five patients were recruited. Twenty six patients completed the 16 week follow-up (figure 1). The baseline clinical characteristics of these 26 patients are represented in table 1. Thirteen patients were Caucasians with 6 South-East Asians, 6 Afro-Caribbeans and 1 of Chinese origin. Six patients had a family history of ischaemic heart disease. Three patients had a history of ischaemic heart disease, but none had suffered a myocardial infarction (MI) or stroke in the preceding 3 months.

**Table 1:** Characteristics of the study population at baseline (n = 26).

Variables	Mean± 1SD or n(%)
Age (years)	50±14
Males (%)	19 (73%)
**Ethnicity (%)**	
Caucasian	13 (50%)
South-east Asian	6 (23%)
Afro Caribbean	6 (23%)
Chinese	1 (4%)
Hypertension (%)	22 (87%)
Current Smokers (%)	6 (23%)
Hyperlipidaemia (%)	9 (36%)
Family history of CVD (%)	6 (23%)
History of CVD (%)	3 (11%)
MDRD estimated GFR (ml/min/1.73 m^2^)	41±11
**Treatment**	
ACEi or AT II RA	20 (77%)
Statins	11 (42%)
Aspirin	4 (15%)
Betablockers	4 (15%)
Nitrates	0 (0%)

**Legend:** Baseline demographics and characteristics of study population that completed the study: MDRD eGFR: Abbreviated four variable Modified Diet of Renal Disease estimated Glomerular Filtration Rate^48^. Mean automated sitting clinic blood pressure taken thrice using appropriate size cuff was measured. Hypertension was defined as clinic blood pressure of ≥140/80 mmHg or on antihypertensives medications. Diabetes Mellitus was defined by WHO guidelines 2006 for definition of diabetes. Hyperlipidemia was defined as Total cholesterol >5 mmol/L and/or LDL>2.5 and or HDL<1.55 or on lipid lowering therapy (National cholesterol Education Program ATP III guidelines 2001). ACEi- Angiotensin converting enzyme inhibitor, AT II RA- Angiotensin II receptor antagonist.

There was no significant change in the number of patients on angiotensin converting enzyme inhibitors or angiotensin receptor blockers or their dosages during the study.

All patients had a low baseline 25(OH)D level with a mean level of 43±16 nmol/L; range 13–74 nmol/L (normal reference range 75–200 nmol/L). The calcium levels were within normal limits 2.37±0.09 mmol/L (normal reference range 2.2–2.6 mmol/L). The parathyroid hormone levels were high with a mean of 10.8 pmol/L, range 3–41 pmol/L (normal reference range 1.1–6.9 pmol/L).

At the end of 16 weeks vitamin D concentrations improved from 43±16 to 84±29 nmol/L, p<0.001. The calcium increased (2.37±0.09 to 2.42±0.09 mmol/L; p = 0.004); and the parathyroid hormone decreased (10.8±8.6 to 7.4±4.4; p = 0.001). The estimated GFR (eGFR) and systolic and diastolic blood pressure remained very stable.

### Impact on Vascular function

**Figure 2:**
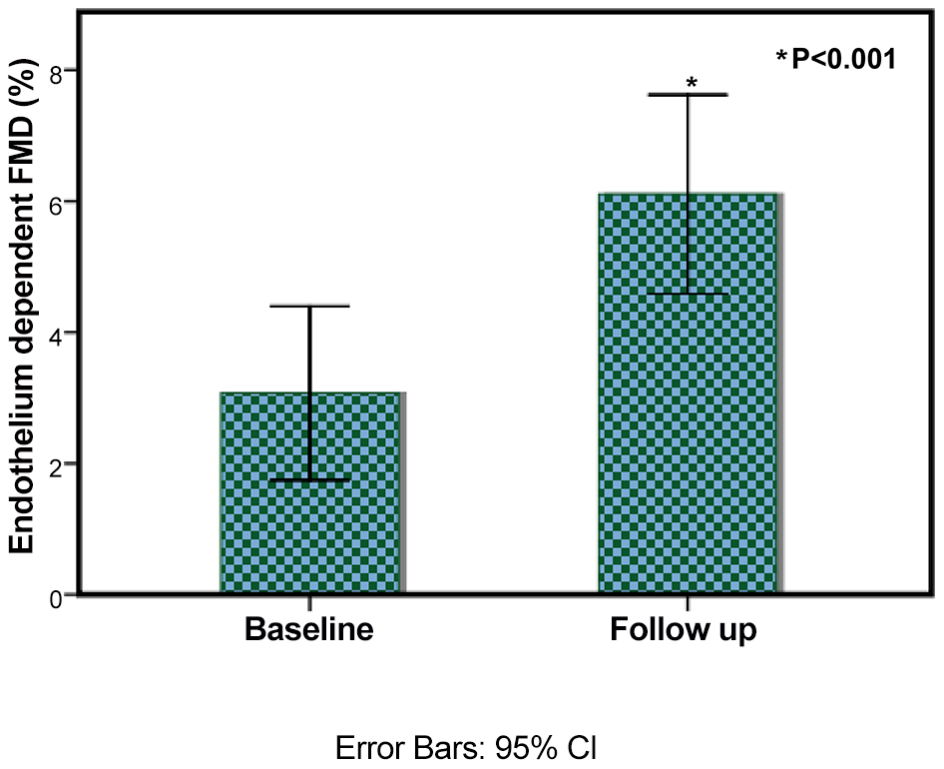
Measure of endothelial function before and after Vitamin D Therapy. Legend: The endothelial function as measured by brachial artery flow mediated dilatation improves from 3.1±3.3% to 6.1±3.7%. Error bars show 95% CI, p<0.001.

The brachial artery FMD improved from 3.1±3.3% to 6.1±3.7%, p<0.001 (figure 2). The PWV and AI tended to improve but did not reach statistical significance, as shown in table 2. There was no significant change in haemoglobin or haematocrit values during the study.

**Table 2:** Analysis of baseline and follow up parameters after Cholecalciferol therapy.

Variables (n = 26)	Baseline (week 0)	Follow up (16 weeks)	p value
Haemoglobin (g/dl)	14.1±1.8	13.8±6.7	0.379
Haematocrit (%)	44±6	43±5	0.486
Serum Albumin (mmol/L)	36±6	38±4	0.283
Urine Protein Creatinine Ratio (mg/mmol)†	35.7±124.2	30.15±221.3	0.866
Serum 25 (OH)D (nmol/L)	43±16	84±29	<0.001*
Serum Calcium (mmol/L)	2.37±0.09	2.42±0.09	0.004*
Serum Phosphate (mmol/L)	1.07±0.20	1.10±0.19	0.459
Serum Parathyroid hormone (pmol/L)	10.8±8.6	7.4±4.4	0.001*
MDRD eGFR (ml/min/1.73 m^2^)	41±11	40±12	0.559
Systolic Blood Pressure (mmHg)	133±12	133±17	0.991
Diastolic Blood pressure (mmHg)	87±9	85±10	0.309
Central pulse pressure (mmHg)	33±12	35±14	0.349
Baseline brachial artery diameter (mm)	4.6±0.9	4.6±0.7	0.945
Endothelium dependent FMD (%)	3.1±3.3	6.1±3.7	0.001*
Endothelium independent FMD (%)	7.34±4.5	10.38±6.7	0.121
PWV (m/s)	7.9±1.9	7.7±2.2	0.059
AI (%)	22±16	18±20	0.055
On ACE I or AT II RA [n(%)]	20(77%)	20(77%)	NC
On Statin	11(42%)	11(42%)	NC
On Nitrate	0%	0%	NC
HsCRP (mg/L)†	3.35±4.75	4.00±4.42	0.272
E selectin (pg/ml)	5666±2123	5256±2058	0.032*
ICAM (ng/ml)	3.45±1.01	3.10±1.04	0.038*
sVCAM 1 (ng/ml)	54±33	42±33	0.006*
vWF (mU/ml)	23.7±12.2	21.6±12.2	0.076
FGF-23 (RU/ml)	131±81	132±67	0.862

**Legend:** MDRD eGFR: Abbreviated four variable Modified Diet of Renal Disease estimated Glomerular Filtration Rate (REF). Mean automated, sitting, clinic, blood pressure taken thrice using appropriate size cuff was recorded.

* denotes p<0.05.

† Distribution non-parametric variable represented as median±IQR, Wilcoxon Signed Rank Test used to analyse the difference in distribution of baseline and follow up values.

ACE I- Angiotensin converting enzyme inhibitor, AT II RA- Angiotensin II receptor antagonist. NC: No Change.

### Impact on blood biomarkers

The circulating biomarkers for endothelial function also showed an improvement. The E-Selectin decreased from 5666±2123 to 5256±2058 pg/mL, p = 0.032. The ICAM-1 levels decreased from 3.45±1.01 to 3.10±1.04 ng/mL, p = 0.038. The VCAM-1 level decreased from 54±33 to 42±33 ng/mL, p = 0.006. The vWF levels improved, but the change was not statistically significant 23.7±12.2 to 21.6±12.2 mU/mL, p = 0.076. There was no change in hsCRP concentrations or urine protein creatinine ratio. There was no change in FGF-23 levels before and after vitamin D therapy (131±81 vs. 132±67 RU/ml; p = 0.862).

## Discussion

The present study demonstrate that supplementation with cholecalciferol in stable, moderate CKD was associated with improvement of vascular endothelial function. Vitamin D3 administration over 16 weeks almost doubled the vitamin D levels, and this was associated with an appropriate decrease in parathyroid hormone levels. The calcium levels, increased slightly, but none of the patients developed hypercalcaemia (serum calcium >2.60 mmol/L). There were reductions in concentrations of circulating biomarkers of endothelial dysfunction, E-selectin, ICAM-1 and VCAM-1. There were no changes in hsCRP, FGF-23 levels and arterial stiffness over the 16 weeks.

In the present study we have measured 25(OH)D, and not 1,25 di-hydroxy vitamin D [1,25(OH)_2_D], because studies have shown that the deficiency is reliably related to low 25(OH)D levels and measurement of 1,25(OH)_2_D levels is challenging, unreliable and expensive and therefore not available to most clinicians [25]. Furthermore, 25(OH)D is inexpensive, easy to administer and has few side effects, even when administered in large doses. Moreover, studies have shown that 25(OH)D levels do stimulate Vitamin D receptors, with less affinity but with higher availability for binding due to its 1000-fold higher concentration in serum [26], [27]. There have been no reports of hypercalcaemia with high dose vitamin D supplementation in this population, and this was also true in our study [28].

Vitamin D is an excellent target for treatment in the context of CKD because of its association with CV disease and endothelial function. In the general population, vitamin D deficiency is associated with increased all-cause mortality, as well as CV event rates [28], [29]. In patients with CKD, relationship of low vitamin D with mortality and CV event rate have been demonstrated in both dialysis and pre-dialysis patients [15], [30]. We and others have previously demonstrated an association of 25(OH)D deficiency with endothelial function [14]. However the present study was the first to investigate the impact of cholecalciferol therapy on endothelial dysfunction in vitamin D deficient/insufficient pre-dialysis CKD patients.

Endothelial dysfunction is a harbinger of atherosclerosis. It is often the first abnormality demonstrated which can predict the initiation and progression of atherosclerotic artery disease [4]. We and others have demonstrated highly abnormal endothelial function in CKD, associated with increased carotid intima-media thickness, hence it is a possible mechanism of initiation and progression of atherosclerosis in CKD [5]. In the general population, endothelial dysfunction within coronary arteries, predicts future CV events [3]. In large general population studies, endothelial dysfunction measured by brachial artery FMD predicts both CV events and mortality [9]. In CKD patients, endothelial dysfunction is an independent predictor of adverse CV outcomes [31].

Endothelial function measured in the peripheral arteries has been used in different patient groups to study the effect of vitamin D supplementation on vascular function. Amongst several such studies which we could identify, three studies demonstrated an improvement of endothelial function with cholecalciferol therapy but the rest did not. In overweight African-Americans the flow mediated dilatation improved by 1.8%; in post stroke patients with FMD improved by 6.9%, and in diabetic patients the FMD improved by 2.3% [18], [32], [33]. The studies where vitamin D therapy was unable to show improvement often used low dose vitamin D supplementation; such as 1,000 units per day for two weeks in a randomised control trial of diabetic patients; and 2,500 IU units per day for four months in post menopausal women [34], [35]. It is conceivable that these studies were unable to show a benefit as the dose of vitamin D used was physiologically inadequate. In other negative studies, vitamin D was supplemented even when the baseline levels were within normal limits, for example, in the studies involving post MI and type 2 diabetic patients, serum 25(OH) D concentrations up to 100 nmol/L at baseline. Supplementing 25(OH)D3 may not be as effective in these patients, as supplementing in patients with low 25(OH)D concentrations [36], [37]. Our study included patients with low 25(OH)D and the treatment dose was high.

Impact of Vitamin D on circulating biomarkers of endothelial function has been variable. In post-MI patients or with coronary artery disease, E-selectin did not improve with vitamin D, whereas, in diabetic patients it did [35], [37], [38]. Our study showed improvement in E-selectin, VCAM-1, ICAM-1 together with the improvement in FMD, hence confirming our findings on in-vivo vascular endothelial function analysis.

CKD patients have immune dysregulation [39], [40]. This results in an increased predisposition to infections as well as chronic systemic inflammation adversely affecting the CV system. Vitamin D has pleiotropic effects on the immune system with evidence that it modulates the adaptive immune system. We know from experimental studies, that vitamin D down regulates Th1 activity, dendritic cell maturation and up regulates Th17 regulator activity, thereby possibly suppressing the low grade inflammation in the blood vessel and the heart [41]–[43]. The present study demonstrated improvement in circulating biomarkers of endothelial function but did not investigate the mechanism any further.

This study did not demonstrate any change of FGF-23 concentrations with Vitamin D therapy. FGF-23 concentrations increase with kidney failure and elevated FGF-23 has been associated with abnormal endothelial function, adverse cardiovascular outcomes, and has recently shown to cause left ventricular hypertrophy [44]. Vitamin D therapy has usually been shown to lead to increases in FGF-23 concentrations in CKD and dialysis, though this effect is not consistent; in our repletion study, we did not see any effect on FGF-23 concentration [45]–[47].

This study has demonstrated that abnormal vasomotor and secretory endothelial function can be rapidly improved with 600,000 IU of Cholecalciferol. There were no adverse changes in arterial stiffness. However, 16 weeks is too short a time period to exclude the possibility of later with vitamin D therapy. The rise in serum calcium concentration was very modest, but was statistically significant.

A limitation of this study is that it was conducted over a short period; so long term effect of vitamin D therapy cannot be assessed. Logistic reasons precluded randomisation; and potential bias cannot be excluded. However the study demonstrates improvement of both endothelial vasomotor and secretory function with cholecalciferol i.e there were improvements of flow mediated dilatation as well as the levels of VCAM-1, ICAM-1 and E-Selectin without adverse effects on arterial stiffness and FGF-23. These findings strongly suggest an effect of Vitamin D on the endothelial cells and potentially will lead to further studies investigating the mechanisms of such benefits of cholecalciferol on vascular function. The study excluded patients with CKD due to diabetes; so in this particular patient cohort, which forms a large proportion of the patients we see in our clinics, these results cannot be extrapolated. The study was restricted to pre-dialysis stage 3/4 CKD; so in patients with early CKD (stages 1 and 2), post kidney transplant and dialysis patients the impact of vitamin D supplementation on endothelial function remains unknown. A randomised control trial with a longer duration of follow up is required to confirm the present findings and plan future clinical outcome driven trials.

In conclusion, this study for the first time demonstrates that Cholecalciferol improves endothelial vasomotor and secretory function, in stable non-diabetes stage 3/4 CKD patients without any significant effect on arterial stiffness, calcium and FGF-23 levels.

## Supporting Information

Checklist S1 **CONSORT Checklist.**
(DOC)

Protocol S1 **Trial Protocol.**
(DOCX)
