# Supplementary material for: Impact of Vitamin D Supplementation on Arterial Vasomotion, Stiffness and Endothelial Biomarkers in Chronic Kidney Disease Patients
Source: PLoS One. 2014 Mar 19;9(3):e91363. doi: 10.1371/journal.pone.0091363 (PMC3960127; doi:10.1371/journal.pone.0091363)
Supplement: Protocol S1 — Trial Protocol. (DOCX) [file pone.0091363.s002.docx]

Impact of Vitamin D supplementation on Endothelial Function and CD4^+^CD28^null^ T cell in patient with Chronic Kidney Disease

**PROTOCOL - PROPOSED RESEARCH PROJECT**

1. **PURPOSE:** CKD is a major public health problem with an estimated prevalence of up to 11% in the general population and consuming 2% of overall NHS budget^1^. CV disease is a significant cause of morbidity and mortality in CKD accounting for 50% of all deaths. Recent reports suggest vitamin D deficiency is associated with increased CV mortality in CKD patients^2,3^. NICE 2008 guidelines however do not recommend routine estimation of vitamin D in these patients due to lack of evidence on benefit from randomised control trials. Vitamin D is only prescribed in late stages of CKD, primarily to improve bone profile.

The focus of this proposal will be to study the effect of vitamin D on non traditional CV risk factors in CKD patients. Two specific areas of research are of interest. We will investigate whether a novel subtype of T lymphocytes, CD4^+^CD28^null^ cell, are present in patients with vitamin D deficiency and do they show the killer phenotype. We will also investigate if vitamin D supplementation improves endothelial function and reduces the CD28^null^ cell numbers. We have submitted a grant application to NIHR to study vitamin D treatment and endothelial function. The role of CD4^+^CD28^null^ cells was not included in the application and will be the main objective of the current proposal. Data obtained from the two proposed studies are of value independently but will complement one another.

We have carried out a preliminary study that showed CD4^+^CD28^null^ cells, reactive to human heat shock protein 60 (hHSP60) were present in 3 out of 12 CKD patients studied. The study needs to expand. We have reported that these cells are also present in patients with acute coronary syndrome and have the ability to target autologous tissue engaging in cytotoxic killing.^4^ Whether vitamin D deficiency results in increased CD4^+^CD28^null^ cell numbers needs to be addressed. If treatment with vitamin D is proven to be effective at improving endothelial function and reducing the numbers of these autoreactive cells, routine vitamin D therapy even in early stages of CKD can potentially result in prevention of 288,000 CV events and 12,000 deaths per year, saving money on hospital admissions and management.

**B. BACKGROUND**:

**i. Magnitude of vitamin D deficiency**: CKD is a significant risk factor for vitamin D deficiency. A prospective study of 43 CKD and 103 haemodialysis patients, found 25 hydroxy vitamin D [25(OH)D] deficiency in 86% and 97% of patients respectively^5^. In a pilot study, we have shown Vitamin D deficiency in 60% of CKD patients^6^.

**ii.** **Vitamin D deficiency and CV disease:** The Vitamin D endocrine system is ubiquitous in human tissues. Apart from well recognised action on bone metabolism, Vitamin D has been shown to improve blood pressure, myocardial contractility and reduce thromobogenesis^7^. Vitamin D receptors are expressed in vascular endothelial and smooth muscle cells, suggesting human vasculature as an important target for vitamin D. In patients with ischaemic heart disease, low vitamin D was associated with increased CV mortality (Hazards ratio 2.2, 95% CI 1.6-3.1)^8^. Numerous observational studies in CKD patients have also shown an increase cardiovascular mortality with vitamin D deficiency (OR=1.9), which improves on supplementation^2,3,9,10^.

**iii. Effect of Vitamin D on non-traditional CV risk factors**: Vitamin D reduces expression of inflammatory cytokines TNF-α, IL-1, IL-6 and IL-12 and upregulates anti-inflammatory cytokine such as IL-10^11^. It has been shown to reduce systemic inflammation in human trials^12^. Furthermore, VDR is expressed on all immune cells particularly antigen presenting cells (APC) and T cells. Active vitamin D induces T-cell differentiation towards Th2 phenotype and by modulating APC maturation and activation, induces T cell anergy and apoptosis^7^. This immune mediation may lead to beneficial effects of vitamin D on non traditional CV risk factors. However no studies have evaluated the impact of vitamin D therapy on vascular function in CKD, a group which is likely to derive the greatest therapeutic benefit with vitamin D supplementation. There are also no studies relating to vitamin D deficiency and presence of CD4^+^CD28^null^ cells. This is a novel area of research.

**iv**. **Preliminary work:** Our previous work has shown a high prevalence of Vitamin D deficiency in our CKD patients, particularly in the ethnic minority. We have also shown a strong association of endothelial dysfunction with inflammation in patients with CKD. Endothelial dysfunction was also associated with increased atherosclerosis in our patients as measured by carotid intima media thickness^13^. We have also shown an increase in CD4^+^CD28 ^null^ cells in patients on haemodialysis (unpublished data). These are an unusual population which carry activatory killer cell immunoglobulin receptors in the absence of the controlling inhibitory counterparts placing the cells in a dysregulated position. Between three hospital sites we have a large CKD population under regular follow up and can therefore reach our recruitment targets.

**C.** **PLAN OF INVESTIGATION**:

**Aims: a)** 25(OH)D deficiency is associated with inflammation and increased CD4^+^CD28 ^null^ T cell population. **(b)** Oral 25(OH)D supplementation in patients with vitamin D deficiency reduces inflammation and CD4^+^CD28 ^null^ T cell numbers. **(c)** Oral 25(OH)D supplementation improves endothelial function in stage 3-4 CKD.

**1. Study population**

- CKD patients between 18 – 75 years, with an estimated GFR (using MDRD formula) 15-60 mL/min/1.73m^2^ will be recruited from the CKD clinics at St George’s, Kingston and Guy’s hospitals.
- Patients with the following conditions will be excluded from the study: Diabetes, malignancy, heart failure, active inflammation, active autoimmune conditions, recent acute coronary syndrome(within last 3 months), rapidly deteriorating renal function, serum calcium> 2.55 mmol/L and if already on Vitamin D supplementation.

**2. Methods: 54 vitamin D deficient patients (25(OH) D levels< 50 nmol/L)** **will be recruited over 10 weeks** **after informed consent.** Haemoglobin, bone biochemistry including PTH, 25(OH)D, circulating CD4^+^/CD28^null^ cells and biomarker of inflammation (highly sensitive C-Reactive Protein) will be measured at baseline along with functional assessment of endothelial function using brachial artery Flow Mediated Vasodilatation (FMD).

**Patients will receive 300,000 IU Colecalciferol orally at 0 and 8 weeks. This dose has proven to be effective in our local population and is safe in patients with CKD^14, 15^ .**

- **Over 16 weeks, mean 25(OH)D level >75 nmol/L will be achieved.**
- **All the above parameters will be studied again at 16 weeks.**

**3. Endothelial Function Measurement:** FMD is a well validated, non-invasive ultrasound test used to assess the integrity of vascular endothelium by measuring endothelium nitric oxide induced vessel dilatation in response to forearm ischaemia^15^. Sublingual Glycerine trinitrate (50 mcg) will be administered to test subjects and further imaging performed for 5 minutes to determine endothelium independent FMD. All measurements will be performed under standardised conditions developed by our unit.

**4. Measurement of Vitamin D:** Serum 25(OH)D will be measured using isotope-dilution liquid chromatography– tandem mass spectrometry. This is routinely carried out at Northwick Park Hospital. Funding for this is available.

**5**. **CD4^+^CD28^null^ cells:** The presence and numbers of circulating CD4^+^CD28^null^ cells will be ascertained by Flow cytometry. Antigen specificity and cytotoxic potential of the cells will be assessed as previously published by our group.^4,16^

**6.** **Statistical analysis**: **Our pilot study has shown FMD to be 3.3± 2.4% in patients with CKD^13^. Assuming a 36% improvement in FMD values with vitamin D therapy (based on a recent trial in type II DM patients)^17^, 51 patients will be necessary for the proposed study (α 0.05 and β 15%). The frequency of CD4^+^CD28^null^ between CKD patients with low vitamin D levels and normal levels of vitamin D will be compared using paired Student’s t-test. Univariate and multivariate analysis will determine effect of vitamin D supplementation on inflammation, CD4^+^CD28^null^ cells and endothelial function.**

**7. Expected Duration: 150 CKD patients/ week are seen at the three hospital sites. 20% of the patients are diabetic and 75% of the patients have a GFR< 60 mL/min/1.73m^2^. Assuming vitamin D deficiency in 60% of our patients, with a recruitment rate of 10%, 54 patients will be recruited over 10 weeks. Repeat analysis will be performed after 16 weeks of Colecalciferol therapy,. With the short duration of the study and continued follow up of patients in the outpatient clinics, we anticipate >95% retention rate of the trial participants. The total duration of the trial will be 27 weeks.**

**References:**

1.National Service Framework for Renal Services. Department of Health; Feb 2005. www.dh.gov.uk/en/Healthcare/Renal/DH_4102636

2. Inaguma D, et al. Clin Exp Nephrol 2008; 12(2): 126-131

3. Wolf M, et al. Kid Int 2007; 72:1004–101.

4. [Zal, B, et al.](http://www.ncbi.nlm.nih.gov/entrez/query.fcgi?db=pubmed&cmd=Retrieve&dopt=AbstractPlus&list_uids=14993140&query_hl=4&itool=pubmed_docsum) Circulation. 2004; 109:1230-1235.

5. Gonzalez EA, et al. Am J Nephrol 2004; 24 (5): 503-10

6. N Chitalia, et al. South West and East Thames Kidney Society Annual Conference; April 2009

7. Bouillon R, et al. Endocrine Reviews 2008; 29:726-776.

8. Dobnig H, et al. Arch Intern Med 2008; 168:1340–1349

9. Teng M, et al. J Am Soc Nephrol 2005;16(4):1115-25.

10. Kovesdy CP, et al. Arch Intern Med 2008; 168:397–403

11. Zittermann A,et al. J Mol Aspects Med 2008; 29: 423-432

12. Timms PM, et al. Q J Med 2002; 95:787–796

13. Banerjee D, et al. J Am Soc Nephrol 2008, 19: Pub 132.

14. Leventis P, et al. Scand J Rheumatol. 2009 Mar-Apr;38(2):149-53

15. Chandra P, Binongo JN, Ziegler TR, et al.. Endocr Pract 2008; 14(1):10-7

16. Zal, B, et al. J Immumol 2008; 181: 5233-41.

17. Sugden JA, et al. Diabet Med 2008; 25:320–325
